# TdfH selectively binds metal-loaded tetrameric calprotectin for zinc import

**DOI:** 10.1038/s42003-022-03039-y

**Published:** 2022-01-31

**Authors:** Aloke K. Bera, Runrun Wu, Simone Harrison, Cynthia Nau Cornelissen, Walter J. Chazin, Nicholas Noinaj

**Affiliations:** 1 Department of Biological Sciences, Purdue University, West Lafayette, IN 47907 USA; 2 Interdisciplinary Life Science - PULSe, Purdue University, West Lafayette, IN 47907 USA; 3 Departments of Biochemistry and Chemistry, and Center for Structural Biology, Vanderbilt University, Nashville, TN 37240 USA; 4 Center for Translational Immunology, Institute for Biomedical Sciences, Georgia State University, Atlanta, GA 30303 USA; 5 Purdue Institute for Inflammation, Immunology and Infectious Disease, Purdue University, West Lafayette, IN 47907 USA

**Keywords:** Pathogens, Cryoelectron microscopy, Bacterial infection

## Abstract

To combat nutritional immunity, *N. gonorrhoeae* has evolved systems to hijack zinc and other metals directly from host metal-binding proteins such as calprotectin (CP). Here, we report the 6.1 Å cryoEM structure of the gonococcal surface receptor TdfH in complex with a zinc-bound CP tetramer. We further show that TdfH can also interact with CP in the presence of copper and manganese, but not with cobalt.

## Abstract (web summary)

The human defense mechanism against pathogens involves limiting essential nutrients, such as zinc which is countered by surface receptor TdfH pirating zinc from human calprotectin. The TdfH cryo-EM structure from N. gonorrhoeae in complex with a calprotectin tetramer provides insights into the protein-protein interaction and the role of metal ions.

## Introduction

**Fig. 1:**
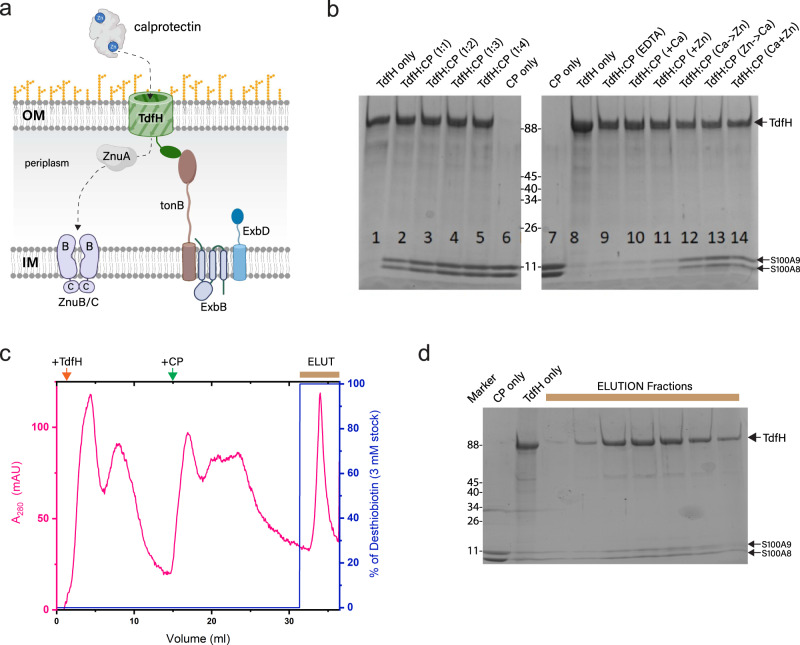
TdfH expression and complex formation for cryoEM studies. **a** The role of TdfH in zinc piracy and import from calprotectin. Figure prepared using BioRender. **b** As a reference, purified TdfH and CP were loaded together with increasing concentrations of CP (lanes 1–6). Complex formation was then performed under various calcium and zinc conditions (lanes 8–14) and pulled down using Streptavidin MagneSphere Paramagnetic Particles. The eluted samples were then analyzed by SDS-PAGE analysis. CP is a heterodimer consisting of one copy each of S100A8 and S100A9. **c** Purification profile of complex formation on an Akta FLPC system using a StrepTactinXT column. TdfH was bound first, followed by CP, and lastly, the complex was eluted with desthiobiotin. **d** SDS-PAGE of the elution peak from panel **c**, verifying TdfH-CP complex formation.

*Neisseria gonorrhoeae* causes the sexually transmitted infection gonorrhea, which can lead to more serious conditions including bacteremia, increased risk of HIV/AIDS, and infertility^1,2^. Due to the rapid emergence of multi-drug resistance, the CDC currently lists *N. gonorrhoeae* at the top of the list of urgent threats to human health, with treatment options now limited to just ceftriaxone. No vaccine option is currently available, intensifying the need for new, more effective antibiotics to combat *N. gonorrhoeae* infection. Recent approaches have targeted virulence factors found on the surface of the pathogen. These well-conserved membrane proteins facilitate pathogenesis by subverting nutritional immunity, which the host uses to combat the proliferation of bacterial invaders by limiting essential nutrients^3–6^. To counter this host defense, *N. gonorrhoeae* has evolved systems that specifically hijack nutrients from the host’s nutritional immunity repertoire for their own survival^7–9^. One such example of this is the zinc transporter TdfH, which has recently been shown to pirate zinc from human calprotectin (CP) (Fig. 1a)^10,11^. CP is composed of two S100 EF-hand calcium-binding proteins, S100A8 and S100A9 that, uniquely among S100 proteins, greatly prefer to form their heterodimer over the corresponding homodimers^12^. As a heterodimer, CP has two distinct transition metal-binding sites at the dimer interface^3,13,14^. Site 2 is a canonical S100 transition metal-binding site with three His and one Asp side chains that coordinates zinc and copper with high affinity. Site 1 is a unique transition metal-binding site composed of six His residues, which along with zinc and copper, is able to chelate Fe(II) and manganese^14,15^. Importantly, CP forms tetramers upon binding of calcium, zinc, copper, or manganese^13,14^. TdfH is a gonococcal TonB-dependent transporter that is zinc regulated and was recently shown to interact with human, but not mouse, CP through both zinc-binding sites^16^. However, the exact details of this TdfH-CP interaction, and the mechanism that enables TdfH to extract and import zinc, remain unknown since no structure that captures this interaction has yet been reported.

## Results and discussion

To better understand the requirements for TdfH to interact with human CP, TdfH containing an N-terminal twin-strep tag was expressed in inclusion bodies, refolded, and purified as described in our previous studies^16^. As a reference to monitor relative binding ratios in our binding assays, TdfH was analyzed by SDS-PAGE with increasing ratiometric concentrations of CP (Fig. 1b; lanes 2–5). TdfH was then bound to Streptavidin MagneSphere Paramagnetic Particles and assayed for binding under various CP buffer conditions. No binding was observed with just calcium or zinc alone, or in the presence of EDTA (Fig. 1b; lanes 9–11). This was surprising since our earlier studies indicated that CP bound to TdfH without exogenous calcium or zinc^16^. We rationalize that because CP binds these metals with high affinity, residual metals may have been picked up from the buffer, enabling this interaction. Experiments reported here demonstrate that both calcium and zinc are required for the stable interaction between CP and TdfH and furthermore that the order of calcium or zinc addition to CP has no effect on the complex formation (Fig. 1b; lanes 12–14).

For cryoEM studies, TdfH from a large-scale prep was purified using a StrepTactinXT column attached to an Akta FPLC automated purification system. TdfH was first flowed over the column followed by CP, washing, and then elution of the complex (Fig. 1c). SDS-PAGE analysis confirmed the presence of both TdfH and CP (Fig. 1d) and the sample was then concentrated and grids were prepared. Data were collected using a Titan Krios equipped with an energy filter and a Gatan K3 detector. Particles were then picked and extracted from ~2,500 micrographs (Supplementary Fig. 1 and Table 1). Iterative rounds of 2D and 3D classification were used to filter particles, resulting in a final 3D reconstruction of the TdfH-CP complex to 6.1 Å resolution using ~12,000 particles.

**Table 1:** CryoEM data collection and refinement statistics.

	*Ng*TdfH-CP
**Data collection and processing**	
Magnification	81,000
Voltage (kV)	300
Electron exposure (e^−^/Å^2^)	44.75
Defocus range (µm)	−1.5 to −2.5
Pixel size (Å)	0.54
Symmetry imposed	C1
Initial particles (Blob Picker) (no.)	2,587,376
Final particles (no.)	11,724
Map resolution (Å)	6.05
FSC threshold	0.143
**Refinement**	
Model Resolution (Å)	6.05
Map-model CC	
CC_mask	0.75
CC_box	0.71
CC_peaks	0.52
CC_volume	0.74
CC_TdfH only	0.69
CC_CP only	0.85
Model Composition	
Non-hydrogen atoms	8117
Protein residues	1115
B factors (Å^2^)	
Protein	190
R.M.S. deviations	
Bond lengths (Å)	0.007
Bond angles (°)	1.149
Validation	
MolProbity Score	3.11
Clashscore	52.49
Rotamer outliers (%)	1.54
Ramachandran Plot	
Favored (%)	77.36
Allowed (%)	22.18
Outliers (%)	0.46
EMDB code	EMD-25692

**Fig. 2:**
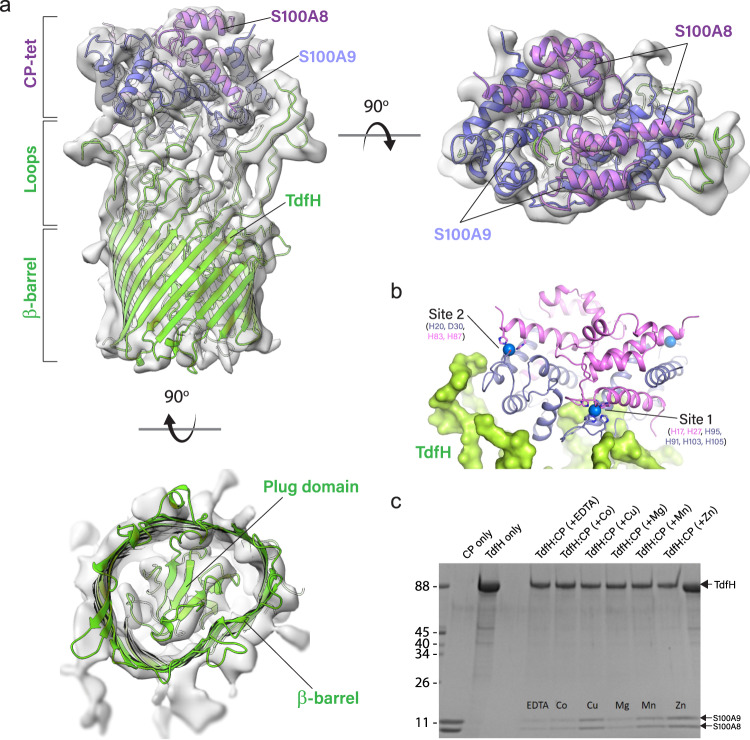
CryoEM structure of the TdfH-CP complex to 6.1 Å resolution. **a** Orthogonal views of the cryoEM map aligned with the TdfH-CP complex structure (TdfH is in green, S100A8 in violet, and S100A9 in lavender). For clarity, calcium and zinc are not shown and the “surface dust” option in ChimeraX was used which removed most of the micelle density. **b** Zoomed view of the interaction of TdfH with site 1 and site 2 of CP, with zinc shown as blue spheres. For clarity, this view is rotated ~180° compared to the view in panel **a** and calcium is not shown. **c** Pull-down assays demonstrate that TdfH is able to bind CP loaded with other metals such as copper (Cu) and manganese (Mn), but not with magnesium (Mg) or cobalt (Co).

Inspection of the final map revealed that TdfH shared the same canonical fold as other TonB-dependent transporters, consisting of a 22-stranded β-barrel domain with an N-terminal plug (Fig. 2a, bottom). Additional density sitting above TdfH was initially modeled as a heterodimer of CP, however, a single heterodimer was insufficient to model the full density available. It was apparent, however, that there was pseudo-twofold symmetry which was consistent with a hetero-tetramer of CP. Therefore, we then performed a rigid fit of the crystal structure of heterotetrameric CP in complex with manganese (PDB ID 4XJK), consisting of two copies of the S100A8-S100A9 heterodimer (Fig. 2a, right). And while the resolution was not sufficient to allow unambiguous modeling of the full TdfH protein, it was clear that the TdfH loops extend up from the core barrel domain to mediate interaction with the CP tetramer. A homology model of TdfH^16^ was then fit into the density and each of the extracellular loops manually adjusted to best fit the cryoEM density. The full structure was then refined in Phenix, producing model-to-map correlation coefficients of 0.69 for TdfH and ranging from 0.82–0.89 for the four chains of the CP tetramer (Supplementary Movie 1 and Table 1). The refined structure revealed that TdfH interacts with the CP tetramer through an interface involving site 1 of one CP heterodimer and site 2 of the other CP heterodimer (Fig. 2b). This demonstrates the requirement of both transition metal-binding sites for the interaction with TdfH and provides some corroboration for our previously reported binding studies which showed that CP mutants defective in zinc binding at site 1 and/or site 2 disrupted their interaction with TdfH^16^. Interestingly, the other two zinc sites of the CP tetramer do not appear to make direct contact with TdfH in our reconstruction. Efforts to improve the resolution of the TdfH-CP cryoEM structure have been hindered by the inability to saturate CP with zinc or other metals, as CP has a high propensity to precipitate out of solution before even achieving a 1:1 (metal:CP) ratio; a ratio of 2:1 would be needed to saturate both metal-binding sites. Our current studies suggest that this is mediated by intermolecular CP–CP interactions, which may require mutagenesis of surface residues to allow saturation of CP with zinc, and subsequently the formation of a more homogenous TdfH-CP complex for high-resolution structural characterization.

Upon discovering that TdfH directly interacts with the metal-binding sites of CP, we assayed the effect of other metals in mediating this interaction. We tested the ability of cobalt, copper, magnesium, and manganese to enable CP binding to TdfH and found that copper and manganese loaded CP could bind to TdfH; however, no interaction was detected between TdfH and CP when the latter was loaded with either cobalt or magnesium (Fig. 2c). While additional studies are needed, these data suggest that in addition to zinc, TdfH could play a role in the uptake of other essential transition metals including copper and manganese.

Our findings in the current study demonstrate that TdfH from *N. gonorrhoeae* selectively binds the zinc-loaded hetero-tetramer of CP, interacting simultaneously with both transition metal-binding sites. These binding properties would ensure the maximal yield of zinc from CP and optimally promote survival of *N. gonorrhoeae* within the human host during infection. We also show that TdfH can bind CP loaded with copper and manganese, hinting at a role for TdfH in the import of these, and possibly other, transition metals.

## Methods

### Expression and purification of CP and TdfH

Calprotectin (CP) was prepared in the absence of calcium or zinc as previously reported and frozen in aliquots at −80 °C^15,17^. TdfH was expressed in inclusion bodies and refolded as described previously with some modifications^16^. Briefly, the *tdfH* gene was subcloned into the pHIS2 plasmid and expressed in BL21(DE3) cells, producing TdfH with an N-terminal 6x His-tag, TEV site, and twin-strep (TS) tags. The cells were harvested, lysed and the lysate was centrifuged at 7000 × *g* for 20 min at 4 °C to spin down the inclusion bodies. The inclusion body pellet was washed three times with 1x PBS supplemented with 1% Triton X-100 and 5 mM ethylenediaminetetraacetic acid (EDTA) pH 7.4, one time with 3 M urea in 1x PBS, and two times with 1x PBS with 5 mM EDTA pH 7.4 using a dounce homogenizer.

To refold TdfH, washed inclusion bodies were resuspended to 5–10 mg/mL in 8 M urea containing 25 mM β-mercaptoethanol (BME) in a dounce homogenizer and supplemented with 0.5% sarkosyl. This was mixed for 15 min at room temperature and then centrifuged for 15 min at 32,000 × *g*. The supernatant was then diluted 60% in refolding buffer [20 mM Tris-HCl pH 8.0, 200 mM NaCl, 10% glycerol, and 0.17% *n*-Dodecyl-B-d-Maltoside (DDM)] and dialyzed overnight at 4 °C against a 20x volume of 1x PBS pH 7.4. The dialyzed sample was centrifuged at 32,000 × *g* for 15 mins at 4 °C and further purified using immobilized metal affinity chromatography using a Ni-NTA column attached to an Atka Purifier (GE Healthcare). Peak fractions were verified by SDS-PAGE and the fractions containing TdfH were combined and the His-tag removed. TdfH was further purified using a Superdex 200 Increase 10/300 GL column (GE Healthcare) in 1x PBS, pH 7.4, 0.02% lauryl maltose neopentyl glycol (LMNG).

### Small-scale pull-down assays measuring CP binding to TdfH

To determine the optimal conditions for complex formation, TdfH in 1x PBS, pH 7.4, 0.02% LMNG was first bound to Streptavidin MagneSphere Paramagnetic Particles (Promega) and washed in 1x PBS, pH 7.4, 0.02% LMNG. CP was then added under the following conditions: (i) 5 mM EDTA (ii) 200 μM CaCl_2_, (iii) 10 μM ZnCl_2_, (iv) incubated first with 200 μM CaCl_2_, then with 10 μM ZnCl_2_, (v) incubated first with 10 μM ZnCl_2_, then with 200 μM CaCl_2_, and (vi) simultaneously with 10 μM ZnCl_2_ and 200 μM CaCl_2_. Lastly, the particles were washed, then the complexes were eluted with 50 mM biotin and analyzed by SDS-PAGE.

### Large-scale complex formation of TdfH-CP for cryoEM

For large-scale complex formation, refolded TdfH in 1x PBS, pH 7.4, 0.02% LMNG was applied and the flowthrough reapplied to a 1 mL StrepTactinXT (IBA Lifesciences) column attached to an Akta FPLC automated purification system (GE Healthcare). The column was washed and then CP (preincubated with CaCl_2_ and ZnCl_2_) was applied and the flowthrough reapplied 2x, the column was washed again, and then the complex was eluted using sample buffer supplemented with 3 mM desthiobiotin and the fractions analyzed by SDS-PAGE. Fractions containing the TdfH-CP complex were then pooled and concentrated to ~4 mg/mL. One limiting factor of this study is our inability to fully saturate CP with zinc, something that became obvious during our cryoEM studies. CP has a high propensity to precipitate out of solution before even reaching a 1:1 (zinc:CP) ratio; a ratio of 2:1 would be needed to fully saturate CP. From analysis of our cryoEM results, this leads to heterogeneity of the complex and limits the number of zinc saturated TdfH-CP particles that can be analyzed.

### CryoEM data acquisition, analysis, and image processing

For grid preparation, the TdfH-CP complex (1.5–3 mg/mL) was applied to glow-discharged (Easiglow, Pelco) Quantifoil R 3.5/1 Cu-200 mesh grids and plunge frozen using a ThermoScientific Vitrobox Mark IV. The cryoEM data were collected on a Titan Krios microscope (ThermoFisher) operated at 300 kV with a nominal magnification of 81,000x using a K3 direct electron detector (Gatan) operated in super-resolution counting mode using Leginon for automated data collection. The images were recorded at a defocus range of −1.5 to −2.5 μm, with a calibrated physical pixel size of 0.54 Å/pixel, with a total dose of 44.75 e^−^/Å^2^; a total of ~3300 movies were collected.

For image processing, motion correction of the movies was conducted on the fly using MotionCor2^18^ with a binning factor of 2 implemented within RELION-3^19^. Using Cryosparc^20^, images were filtered based on CTF-resolution fit, leaving ~2500 images for initial blob picking. Initial templates were prepared by iterative rounds of 2D classification, which were then used for template picking, producing an initial ~2.5 million particles. Further filtering by interactive rounds of 2D classification yielded ~120,000 particles of top and side views, based on clear features including the presence of the detergent micelle. Multiple 3D classes were prepared by ab initio modeling followed by heterogeneous refinement, with only a single 3D class (~63,000 particles) having features consistent with a membrane protein complex within a detergent micelle. The 3D classification was iterated to further filter the particles until a homogenous 3D class was attained. Homogenous refinement was then performed, followed by non-uniform refinement and finally, local refinement to produce the final 6.1 Å reconstruction of the TdfH-CP complex. During the 3D classification, a majority of the particles appeared to be TdfH-only; however, attempts to process these further was unsuccessful due to heterogeneity of the shape of the barrel and the lack of defined features outside of the micelle. We hypothesize that this may be due to the increased flexibility of the barrel and loops of TdfH in the absence of CP.

For model building, the manganese-bound CP structure (PDB ID 4GGF) and our previously reported homology model for TdfH^16^ were fit into the map using ChimeraX^21^. For TdfH, the loops were all removed and manually traced into the visible density available. Given the resolution and lack of sufficient density, not all residues were modeled, which is consistent with other similar structures where many of the large extracellular loops are expected to be partially or fully disordered due to flexibility. The final model was refined using real-space refinement, concurrently performing rigid-body refinement of all the individual chains with secondary structure restraints. All model building was performed using COOT^22^ and real-space refinement was performed using PHENIX^23^. A higher resolution cryoEM structure is needed to unambiguously localize all of the residues of TdfH.

### Small-scale pull-down assays measuring the effect of metals on CP binding to TdfH

To determine the effect of metals other than zinc on complex formation between CP and TdfH, TdfH in 1x PBS, pH 7.4, 0.02% LMNG was first bound to Streptavidin MagneSphere Paramagnetic Particles (Promega) and washed in 1x PBS, pH 7.4, 0.02% LMNG. CP was then added under the following conditions: (i) 5 mM EDTA, (ii) 200 μM CaCl_2_ and 10 μM of CoCl_2_, (ii) 200 μM CaCl_2_ and 10 μM of CuCl_2_, (iv) 200 μM CaCl_2_ and 10 μM of MgCl_2_, (v) 200 μM CaCl_2_ and 10 μM of MnCl_2_, and (vi) 200 μM CaCl_2_ and 10 μM of ZnCl_2_. Lastly, the particles were washed, then the complexes were eluted with 50 mM biotin and analyzed by SDS-PAGE.

### Reporting summary

Further information on research design is available in the Nature Research Reporting Summary linked to this article.

## Supplementary information

Supplementary Information

Description of Additional Supplementary Files

Supplementary Movie 1

Reporting Summary

## Data Availability

The cryoEM map of the TdfH-CP complex has been deposited in the Electron Microscopy Data Bank with accession EMDB code EMD-25692. All other data are available from the authors on request.
