# Supplementary material for: TdfH selectively binds metal-loaded tetrameric calprotectin for zinc import
Source: Commun Biol. 2022 Jan 31;5:103. doi: 10.1038/s42003-022-03039-y (PMC8803948; doi:10.1038/s42003-022-03039-y)

Supplementary Information.

Supplementary Figure. 1. CryoEM pipeline for structure determination of the TdfH-CP complex. a. The reconstruction workflow starting with micrographs, particle picking, iterative 2D classification with filtering, iterative ab initio with hetero refinement, non-uniform refinement, and local refinement. **b**. FSC plot from Cryosparc to 6.1 Å. **c**. Local resolution plot of the TdfH-CP complex structure.

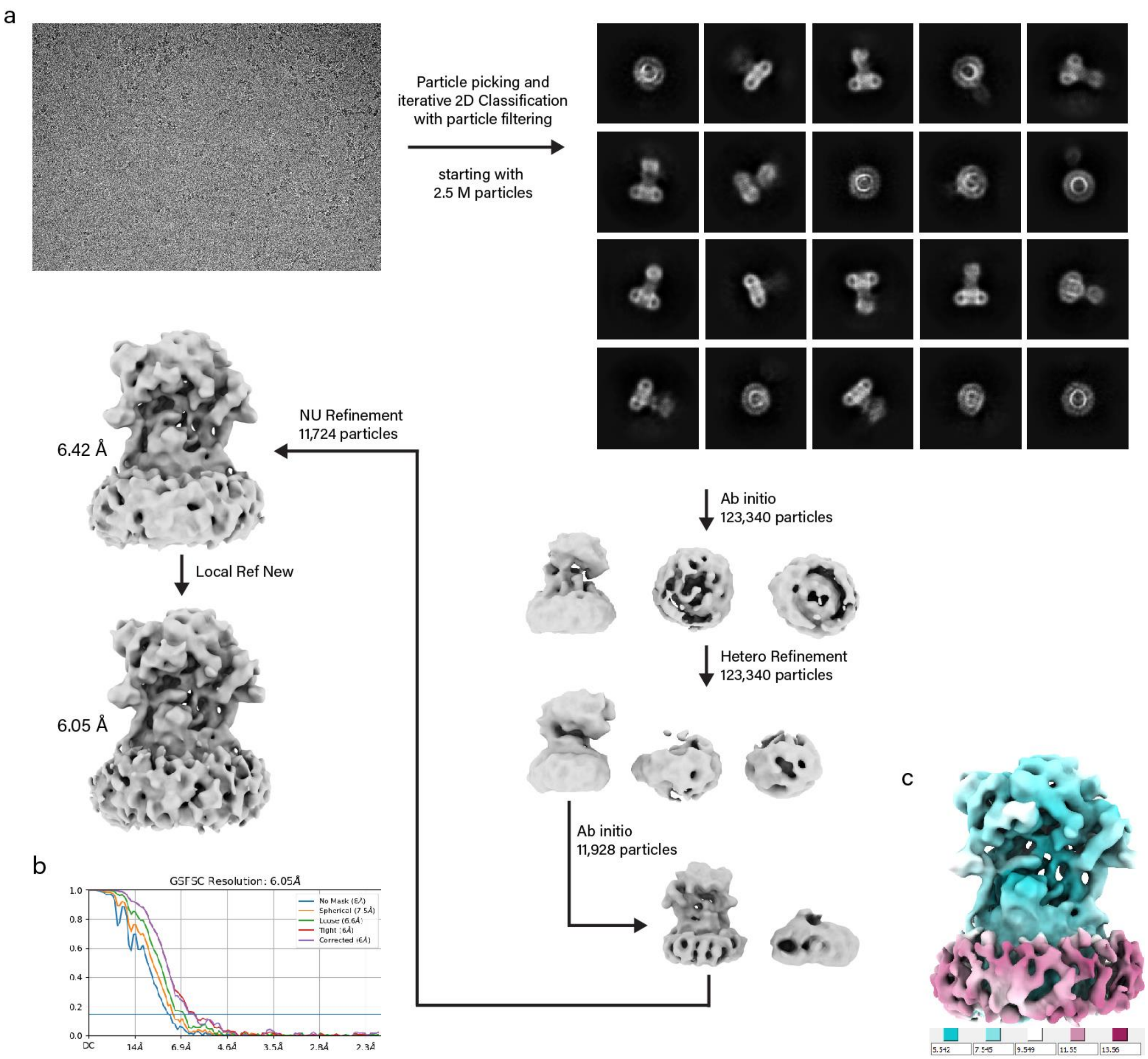

Supplement: Supplementary file 1 — Supplementary Information [file 42003_2022_3039_MOESM1_ESM.pdf]
