# Supplementary material for: TdfH selectively binds metal-loaded tetrameric calprotectin for zinc import
Source: Commun Biol. 2022 Jan 31;5:103. doi: 10.1038/s42003-022-03039-y (PMC8803948; doi:10.1038/s42003-022-03039-y)
Supplement: Supplementary file 2 — Description of Additional Supplementary Files [file 42003_2022_3039_MOESM2_ESM.pdf]

Description of Additional Supplementary Files

File name: Supplementary Movie 1
Description: CryoEM structure of the TdfH-CP complex. A movie of the TdfHCP complex to 6.1 Å resolution highlighting the quality of the density and the fit of the TdfH and CP models into the density.
